# Professional Self-Reliance—Valedictory Address of Prof. Sanborn

**Published:** 1855-05

**Authors:** John E. Sanborn

**Affiliations:** Prof. of Chemistry and Materia Medica in the Iowa Medical Department


### IOWA MEDICAL JOURNAL.

VOL. II. KEOKUK, IOWA, APRIL & MAY, 1855. NO. IV.

### ORIGINAL COMMUNICATIONS.

### PROFESSIONAL SELF-RELIANCE.

Extracts from a Recent Valedictory Address,

BY JOHN E. SANBORN, M. D.,

Prof. of Chemistry and Materia Medica in the Iowa Medical Department.

The hour possesses to you a more than ordinary interest. The dividing line between the glowing past and the radiant future, its crowding emotions are dedicated alike to memory and hope. If you turn to contemplate the scenes in which we have of late been fellow-laborers, you find the landscape colored with the mellow radiance of pleasing, and I trust, delightful associations; or, you gaze upon the future, and the sunshine of the past has already shed upon it, by reflection, something of its own brilliancy and beauty.

To moralize upon the sentiments appropriate to such a "parting of the ways" is one thing; to feel in *the heart* the throb of those sympathies. is quite another. Here, in the crowded assembly, we may fitly call to mind the emotions and contemplations of which the occasion is so richly suggestive; while it is left for your subsequent hours of calm meditation to linger upon the pleasing associations of the present, and for your future life of active usefulness to embody the practical suggestions, which the anxiety we feel may prompt us to utter.

It is, indeed, almost a presumption on my part, that I should essay to give suitable utterance on behalf of myself and my colleagues, to the earnest and vivid emotions, which, at such an hour, come unbiddem to our hearts; and yet it is not unmeet that the occasion should have its interpreter.

As the departing son, passing forth from the scenes of his boyhood lingers upon the threshold, or wandering thence, turns again and again to gaze at the old homestead, as his retreating footsteps "Drag at each remove a lengthening chain," and finally, at the last bend of the road, pauses to take a long farewell of the paternal roof, so you may well pause, ere you quite cut loose from the pleasing associations of the past, before you set sail upon the broad sea of enticing, perhaps perilous activity. Allow me then to devote the hour allotted to us, to a brief consideration of one of the prominent errors to which the physician is strongly tempted, and into which many fall: a line of suggestion prompted and put forth, not with the assumed position of a teacher, but of an earnest and anxious counsellor and fellow laborer.

You remember well that stirring tragedy in which the great dramatist depicts so vividly the culminating eminence and downfall of Cæsar; you recall the scenes in which Brutus and Cassius hold sad counsel together, that the dark shadow of despotism seemed slowly creeping upon the dial of the republic, and both mourned that "The age was shamed, and Rome had lost the breed of noble bloods;" they mutually lament that Cæsar, "A man of such a feeble temper, should bestride the narrow world like a colossus." It was then, that Cassius, half chiding himself exclaims,

> Men at some time are masters of their fate.
> The fault, dear Brutus, is not in our stars
> But *in ourselves*, that we are underlings.

So too, in that wonderful and harrowing delineation of the sad destiny of Othello, we find a similar utterance, from the pious lips of Iago, that inhuman master-piece of duplicity and villany—where he says:

> "'Tis in ourselves, that we are thus and thus."

I would commend the sentiment to you as one of primary importance, in mental growth and culture. It has respect to the tendency to a neglect, on the part of the physician of a determined and persistent self-reliance.

By self-reliance, I mean a disposition which trusts to one's own abilities and resources, rather than to those of others—that mental courage which dares to encounter and grapple with obstacles, and does not, in the hour of necessity, shrink from the obvious duty; not the poor bravado that courts danger, and then perhaps finds itself worsted in the encounter, but the stern fortitude of spirit which does not shrink from the perils in its pathway, but summons up its own manly resources to the conflict; equally removed on the one hand from the rashness that imprudently dares, and on the other, from the timid cowardice that always quails.

The career of every individual, who has any ambition, above being a yielding material, to be moulded and shaped into any plastic form by the random touch of circumstance, who aspires to the building of an enduring character, who sees in the world around him materials, influences and associations of which he must be the master or the slave—the career of such an one partakes essentially of the nature of a conflict. Day and night, year in and year out, goes on the silent, but ceaseless strife. The solitary human heart is thrown into the arena of this world, for the sole purpose of working out its own destiny. The talents which are returned unimproved but bring disgrace, and not credit to him to whose use they have been confided.

In this conflict with the opposing influences, this warfare of every strenuous spirit, which lasts while life endures, and of which the victory can never be pronounced beyond reverse till death disarms the combatant, no element of success is so important as a thorough knowledge of one's own resources, out of which must spring every reasonable germ of self-reliance. To have a judicious confidence in one's condition, to rely fully upon one's resources, is but the result of an adequate knowledge of the capacity of those resources. A thorough self-knowledge then, is the only proper basis for a calm and manly self-reliance.

We need no encomiums upon the sublime beauty of self-reliance. In the very contemplation of it is its own best eulogy. The spirit that can meet with undaunted breast every legitimate conflict, that stands firm in the strength of its own well fortified nature and pure heart, that manfully relies upon the means God has provided, for the performance of the work, the duty God has pointed out, achieves the highest office of manhood, and deserves the loftiest benisons.

Our view of self-reliance involves, it necessitates the existence of judicious *self-culture.* It is of course based upon the possession of qualities worthy of reliance. Else, how shall we distinguish the thoughtful and commendable reliance or confidence that is founded upon a proper estimate of well-trained powers, from the heedless presumption or impertinent audacity that is born and nourished by ignorance? But, the noblest development of this virtue, though most prominent in the more brilliant display of splendid achievements, does not therefore perform there its highest offices, nor does it always in such illustrious examples, exhibit its truest worth. It is the rather in the even tenor of daily life, in the scrupulously conscientious performance of daily duties, often onerous, sometimes repugnant, that such a virtue shines with serener and more uniform lustre. In precisely this line of duty consists one of the prominent excellencies of a good physician, and it is in the discharge of such duties, in the proper province of the medical man, that there is often a tendency to a neglect of a sound self-reliance.

In searching for the causes which may lead to such neglect, we may find perhaps a prominent one in the very necessities attending a medical education. The period of three years must be spent in preparatory medical study; during all this season, the student is constantly employed in collecting and treasuring up medical facts and medical principles, which he must take upon the authority of others, either authors, or his living teachers. Some of these facts he may be able to verify by actual demonstration, such as the mechanical departments of anatomy, and to some extent of chemistry. But, most of his information is on the strength of the assertion of others. His facts are, and must be, for a season, taken entirely on trust—at least, until he can verify them by subsequent experience and observation. This is necessary from the very nature of the case, and can in no way be avoided. It is a necessity which applies to medical science much more largely than to law or theology; for in these studies the investigation refers chiefly to the application of *principles*, and not to the reception and application of *facts*; principles, which, if true, recommend themselves at once to the common sense of the very learner, and he is therefore never compelled to follow blindly the authority of any teacher and of course never should. Nor should the student of our own profession, after he has once obtained that range of view, which will enable him to comprehend a wider relation of influences and to "Judge of his own self what is right." It is plain, however, from your own experience and from the very necessities of the case, that elementary instruction in medical science can be obtained only by a temporary trust in the absolute authority of others.

Now, it is plain that this temporary exigency of a season, should as much as possible, be thrown far from us, when the period for its employment has once passed. But, the danger is, that this temporary necessity may become in the time a permanent despotism of habit. Have a care, young gentlemen, least the thread with which you at first guide your unwonted feet in the new pathway, shall become by your own submissive consent, a shackle of steel, chaining you to the dull and narrow routine of your earliest footsteps. That is indeed a sad history, in which it is evident that the voluntary attitude for a brief season, becomes the cramping torture of a life time.

Let us be understood; not that you are to avoid reliance upon authorities at any period of your professional pupilage; your progress will be largely indebted to your familiarity with the testimony of others, not only during the term of study, so called, and earlier practice, but during the whole period of life-long tuition. No sane man would wish or hope to cut loose from all the accumulated wisdom of the past. You must by no means neglect so valuable a storehouse of truth and of recorded facts. But—and to this point I would solicit your attention—it depends upon you to say what use you will make of this repository of science. Whether you shall be the tame and undiscriminating recipients of the authority of others, adopting now light, and now darkness, taking in at one time intellectual nourishment, at another, poison, or whether by the noble exercise of a God-bestowed endowment, you shall select and reject; hesitate, consider, and decide.

The avocation of a physician is one which of all others, imperatively demands the exercise of independence of thought. The life of a medical man exhibits an ever changing panorama of shifting forms of disease. Scenes are ever passing before him, similar in their elements, like the wide range of external nature; but, as in natural scenery no two are ever found alike. In such a view of the case, how poor is the sanction or tolerance, for an ignorant routine practice! On the contrary, what a challenge does nature fling out, for the exerciee of the best faculties, by every one who may dare to interpret or assist her. The demand for medical assistance is constantly occurring in cases where the physician's guide should be judgment, not memory; a careful deliberate decision as to what the case in question demands, *not* a blind following of *any* precedent, similar perhaps only in *some* of its outward manifestations. I know full well the strength of the temptation to follow seeming precedents; to adopt an unthinking and routine practice, and I know too that temptations are to be resisted. Let the records of the past, and the memory of your own experience lead to that highest exercise of manly intelligence, the deduction of broad general principles, to an extensive generalization, and let the principles thus deduced, be faithfully applied to the case under consideration. This can be the only true chart, to guide you over the shifting currents and changing quicksands of your arduous voyage. It is evident then how vitally important is such an independence of thought, as must be developed by a rigid self-reliance. While it is indispensable in the daily duties of the physician; it is often called out into still bolder relief and exercise in some instances of sudden and perilous emergencies, when the destiny of not one life only, but of a beloved family circle, perhaps of a still wider range, all seem to hang upon the movements of one individual, and that too in a mere fragment of time. Upon his self-possession and self-reliance depend the interests, the most sacred sympathies of his fellow beings. Such exigencies occur too, not only suddenly, but in some hour and place of loneliness, where the aid and counsel of others cannot be obtained. If you then have within you a mine, from which to draw the treasures of practical knowledge and available principles, it will be an easy task, indeed, a pleasure to make and witness the successful application of well-founded principles. But, upon any other course, how full of shame and disaster must be such an encounter.

We have contemplated the nature and necessity of self-relying independence of thought. The benefits of such individual training are obvious. It is the benefit which we derive from culture of the soil; the advantage gained from working the mine of hidden ore.

It brings forth the latent wealth of the mind—it developes the intellectual resources, which, but for culture, would have remained unknown and useless.

The benefit indicated is the benefit of athletic muscular action, that developes the brawny arm, the symmetrical frame and the sinewy limbs of mature vigor. It asserts the supremacy of true conviction, the result of individual investigation, over blindly trusting to the guidance of others. He only may be said to *know* a fact in science, or to be thoroughly convinced of the truth of a given principle, who has brought to the question the critical test of his own powers of scrutiny, who has gone through the processes of doubt, investigation and conviction. The only true solidity of mental character, which gives weight to professional opinion, is that derived from individual critical examination. The physician who maintains a given opinion because some great teacher so affirms, is at best but a poor pawnbroker of other mens' theories ; unable to give a reason for the faith that is in him, he is and must be as unstable as the wind. With no deep foundation to his own intellectual character, how can such an one be a reliable guide to others ; how dare he apply upon others those principles which he has not brought to the test of his own critical judgment?

True, you will find it easier in a short sighted and a mechanical point of view, to have others think for you, than to think yourselves ; to believe, than to know. But the solemn question of a life time is one of duty, not ease; your mission on this round globe is the development of vital principles, not a dull search for mere enjoyment.

The process enforced is, in itself the very essence of self-culture, the only true basis of vigorous self-reliance, and it is for this especially, that I commend the virtue in question. The act of faithfully analyzing the opinions of others, that we may separate the true from the false, is the very process by which to gain vigor of mind, and enforce maturity of judgment; the very means with which to conduct such analysis to a safe and just issue. Without self-culture, self-reliance is but a vain pretence; and a conviction of the *need* of independence of thought, an abiding sense that it is in ourselves and not in others that we are thus and thus, is the first step towards the possession of its highest elements. But, we are warned that such promotion of the individual judgment, such implicit reliance upon our own solitary reason, is but sowing in our hearts, the seeds of professional vanity; that over estimating ourselves, we become inflated with the most airy conceit. But, he who comes in daily conflict with contradictory views, and marshals the powers of his own thought to the encounter, will hardly over estimate the abilities with which he thus becomes so practically and frequently familiar. He who has so often been put to the test of severe and perhaps unsuccessful labor, must in time learn so much that is unsatisfactory, respecting his qualifications, that he can hardly be flattered by the brilliancy of his success. Besides, and chiefly, the effect of the rigid mental development acquired, by a careful and systematic training of one's powers, is such as to lead to a profound sense of humility and modesty. He who surveys himself alone, and that superficially, may perhaps be pleased to contemplate the extent of his attainments. But, he who takes a wider range and goes beyond his own self, sees more truly how much yet remains unaccomplished.

A human being, confined within the narrow cavern of his own ignorance, sees himself indeed, as the principal object; but, open his eyes and place him upon the mountain tops of science, and the contrast makes his insignificance almost contemptible.

I may add, as another circumstance, indicating the need of this principle, that a discriminating reliance on one's own powers of thought and investigation, such as I have endeavored to illustrate, is the only satisfactory method by which one can keep distinct, within his own mind, the domains of truth and error. How will you be able to learn, and above all to convince yourselves of the right and the wrong, unless you are your own counsellors; unless you are familiar with the evidence of both sides of the argument, and can satisfy yourselves of the justness of the decision? If you rely on the authority of others, that mere authority, whether right or wrong, does not or *should* not satisfy you. You are not nourished, your intellectual system is not strengthened, by the food that supports him. The course of reasoning that may satisfy his nature, may be not at all adapted or adequate to yours. You are to work out, by hard and toilsome effort, your *intellectual* subsistence, as clearly and certainly as you are to maintain your physical support.

That mind must be brought to a serious fear of alarming destitution, that is willing to beg, from volume to volume, the daily food of its mental nourishment; and, I may add, that is content to receive, with the passive hand of charity, whatever medical theories or professional dogmas, others may choose to impose upon it.

It is a fortunate circumstance that in this effort of the individual mind to learn the truth, it need not put itself to the constant torture or anxiety of original experiment, except in fields absolutely new. Happily, the observation which is afforded us of the experience of others, may amply suffice our need; and well is it for any one who can successfully substitute for his own experience, bitter and stumbling, an appreciating knowledge of the experience of others.

But, while we take as our own, and profit by the experience, the researches, the facts of others, let us be careful how we adopt and sanction the opinions which others may deduce from those facts. These are to be rigidly put to the test. If false, to be of course discarded; and even if true, to be doubted till proved true.

It is largely true, as was said by Aristotle, that "incredulity is the source of wisdon." There is hope of him who doubts. He will in good time be one of those who can "give a reason." It cannot be too strongly impressed upon you, that in the world of art and science, scepticism is no crime. It is the fulcrum upon which the lever of reason can almost move the world. By doubt, you investigate, explore, discover and prove. By blind contented credulity, you put out the lights of science, enclose the world in darkness, blind our eyes, and cloud our footsteps.

It cannot be necessary that I should define the province and limitations of doubt; indispensable as a means, fatal as an end; a most useful and healthful avenue, but a pestiferous abode. However desirable may be the mental activity it involves, better by far to limp along depending on external aid, than to petrify in the stagnation of eternal inertia, or to chafe away one's existence in the unending circle of indecision. It is well said,

'Tis a base
Abandonment of reason, to resign
Our right of thought.

But, may it not be an error to regard it as merely a right? I

would that you esteem it the rather a duty; a duty, the neglect of which, involves a recreancy to every other trust.

That is indeed a mournful bankruptcy in which the poor delinquent delivers over, to any individual, or to any class, the quitclaim to his own dominion of thought, to the wide range and roam of his unchained reason. It is a transfer common enough, and yet one not generally, clearly and distinctly set forth; no fixed time is usually assigned when the change of ownership takes place, and it is often not easy to say when the contract is fully closed, or what are the precise terms. And yet, how many do as effectually abstain from all independence of thought, as if the legal documents of a transfer had been fully made out, and duly recorded; the very subjects of such despotism not suspecting the bondage, and for this very insidiousness is the danger the more fearful, the more to be guarded against.

I have thus endeavored to illustrate and enforce the necessity and the benefits of an earnest culture of the powers within; such culture as shall enable these powers to be a true and reliable guide to your conduct, and especially to your professional course, in all the intricate paths of your future life; such a course is, as we have seen, the only true basis of real independence of character. Amid the whirlwind of conflicting sentiments and opposing theories, he only stands upon a reliable basis, whose feet are planted on the solid rock of his own judgment.

Let me urge upon you then, while you draw freely from the printed volume, and the living speaker, while you study the changing phenomena around you, and ask the ever-varying phases of disease what is the burden of their searching, while you neglect no source of information, let me urge you to combine, classify, and analyse these several and perhaps contradictory testimonies, and bring all to the test of your own judgment. Let all things instruct you, but let none be your master. Let the humblest of men be your authority for abstract facts, if need be, but suffer not the most elevated to be your authority in opinion. Vindicate thus your manhood, by the exercise of the rights and duties of manhood.

Though your judgment may not be at first, the best in the world, yet it may be, or should be, the best in the world for you. Disown it, if you will, or neglect it by reason of timidity, or indifference, and you erase from your nature the stamp of the Deity; you commit suicide upon your noblest attribute. But trust in it, nourish, use and strengthen it, and it will faithfully reward your confidence. Though possibly it may not at first perform, with the fullest satisfaction its several duties, yet the very exercise in question will give it a strength and solidity you had never dreamed of. You thus nourish, in your earlier years those elements of your nature, that, in mature and advanced life, shall be your best supporter, comforter, and protector.

Gentlemen:—This evening are sundered the cords that have bound you to the associations of a season. Four months have been devoted by us, to the assiduous duties of the lecture-room. Day after day, and week after week, we have exchanged the salutations of kindness, and engaged in the ennobling pursuits that have absorbed the attention of us all. Through the months that are passed, you have been endeavoring to lay up a storehouse of facts and of principles, that shall help to guide your future steps. But, now, the scenes are changed. You, who have of late been pupils, are such no longer; you go forth, a little band, to instruct and influence, silently it may be, but yet to instruct, the several communities in which you may move. For the noiseless eloquence of your deeds, and the silent utterance of your actions, shall ceaselessly speak of you. This night, ere you cross the threshold of the future, let your wise resolves and your steady purpose of right, give to this language of a life-time such tones of harmony and approval, as shall bring joy to many a human heart, and make your pathway vocal with melody.

In your counsels with your own hearts, and in your actions in the outer world, dare to maintain and manifest those principles of right and duty, which have their throne in the heart of every man.

Dare to do all, that doth become a man,

Remembering that he "who dares do more, is none." Have the courage, for it often needs the truest courage, to be loyal to your own convictions of honor.

To thine own self be true;
And it must follow, as the night the day,
Thou canst not then be false to any man.

It is the moral of the hour, that your destiny lies almost exclusively in your own hands; this fact, which to a weak and timid spirit, is a fearful incumbrance, a barrier to its progress, let it be to you but the stimulus in which the valiant soul rejoices. With you is to be alike the achievement of conquest, and with you, if so it must be, the name of inglorious disaster; with you, it is to say whether the medical education thus far shall be regarded as final, or rather as the foundation stones and scaffolding, by aid of which you will erect, slowly and securely, the structure of a complete and useful professional life.

